# Hypothesis: The Role of Sterols in Autism Spectrum Disorder

**DOI:** 10.1155/2011/653570

**Published:** 2011-04-27

**Authors:** Ryan W. Y. Lee, Elaine Tierney

**Affiliations:** ^1^Department of Neurology and Developmental Medicine, Kennedy Krieger Institute, 716 North Broadway Street, Baltimore, MD 21205, USA; ^2^Department of Pediatrics, Johns Hopkins University School of Medicine, 600 North Wolfe Street, Baltimore, MD 21287, USA; ^3^Department of Psychiatry, Kennedy Krieger Institute, 716 North Broadway Street, Baltimore, MD 21205, USA; ^4^Department of Psychiatry, Johns Hopkins University School of Medicine, 600 North Wolfe Street, Baltimore, MS 21287, USA; ^5^Center for Genetic Disorders of Cognition and Behavior, Kennedy Krieger Institute, 716 North Broadway Street, Baltimore, MD 21205, USA

## Abstract

A possible role for sterols in the development of autism spectrum disorder (ASD) has not been proven, but studies in disorders of sterol biosynthesis, chiefly Smith-Lemli-Opitz syndrome (SLOS), enable hypotheses on a causal relationship to be discussed. Advances in genetic technology coupled with discoveries in membrane physiology have led to renewed interest for lipids in the nervous system. This paper hypothesizes on the role of sterol dysfunction in ASD through the framework of SLOS. Impaired sonic hedgehog patterning, alterations in membrane lipid rafts leading to abnormal synaptic plasticity, and impaired neurosteroid synthesis are discussed. Potential therapeutic agents include the development of neuroactive steroid-based agents and enzyme-specific drugs. Future investigations should reveal the specific mechanisms underlying sterol dysfunction in neurodevelopmental disorders by utilizing advanced imaging and molecular techniques.

## 1. Introduction

The autism spectrum describes a group of disorders with early childhood onset, characterized by persistent core deficits in socialization, language, and stereotypic and repetitive behavior [1]. Over 50 years has passed since Leo Kanner pioneered a description of infantile autism [2]. The definition of autism has expanded to include a wide spectrum of clinically and biologically heterogeneous disorders, each with variable degrees of core autistic feature expression, which we now describe as autism spectrum disorder (ASD) [3]. The estimated prevalence of ASD in the United States is 1 in 110 children [4]. The list of well-defined genetic disorders with ASD continues to expand, with commonly studied examples including fragile X syndrome, tuberous sclerosis, untreated phenylketonuria (PKU), Rett syndrome, and Smith-Lemli-Opitz syndrome (SLOS). Thus, studies involving relatively homogenous populations with well-described genetic disorders have begun to reveal the neurobiologic underpinnings of behavioral phenotypes such as ASD. Evidence supporting a role for sterols in the development of ASD was based on studies in disorders of sterol biosynthesis, chiefly SLOS [5–8]. Furthermore, a study of 100 serum samples from the Autism Genetic Resource Exchange (AGRE) demonstrated that a subset (about 20%) of unrelated children from multiplex families with ASD had mild hypocholesterolemia (i.e., lower than 100 mg/dL), which is in contrast to very low cholesterol levels (<10 mg/dL) often seen in severe SLOS cases [6]. The findings of Tierney et al. were replicated when an additional 100 AGRE subjects were tested by the same group (unpublished data), but have not as yet been replicated by other research teams.

Cholesterol serves many essential roles in the developing nervous system. It is a structural component of myelin and membrane lipid rafts, serves as a substrate for neurosteroid formation, and facilitates hedgehog signaling [9, 10]. Impaired function of these activities is likely responsible for the anatomic and neurobehavioral manifestations in SLOS. Recent advances in gene technology and membrane biology have contributed to a better understanding of the complex mechanisms underlying impaired cognition and behavior in cholesterol-deficient conditions. This paper hypothesizes on the role of sterol dysfunction in ASD and proposes future directions for targeted therapeutics. We hypothesize that cholesterol dysfunction may lead to ASD by three mechanisms working in concert during brain development: (1) impaired sonic hedgehog patterning, (2) alterations in membrane lipid raft structure and protein function resulting in abnormal synaptic plasticity, and (3) impaired neurosteroid synthesis.

## 2. Sonic Hedgehog and Cholesterol Dysfunction in SLOS

**Figure 1:**
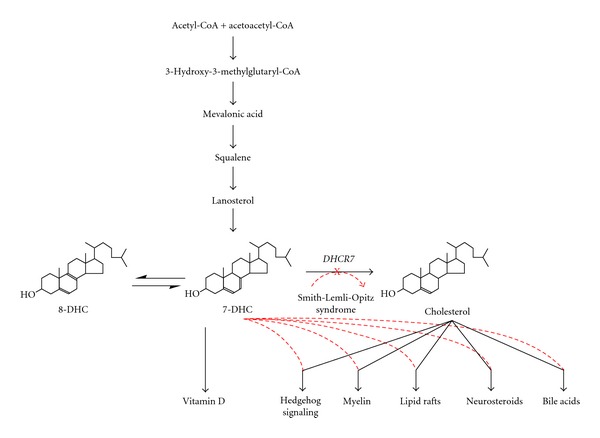
Effect of sterol precursor substitution in Smith-Lemli-Opitz syndrome. (Adapted with permission from Richard Kelley, M.D. and Forbes Porter, M.D.).

Smith-Lemli-Opitz syndrome (SLOS) is an autosomal recessive disorder of cholesterol biosynthesis caused by mutations in the gene encoding 7-dehydrocholesterol reductase (*DHCR7*) located on chromosome 11q12-13 [11, 12] (Figure 1). SLOS has an estimated incidence among individuals of European ancestry of 1 in 15,000 to 1 in 60,000 births and a carrier frequency of 1 in 30 to 1 in 50 [13–17]. Individuals with SLOS have abnormally elevated plasma 7-dehydrocholesterol (7-DHC) or its isomer 8-dehydrocholesterol (8-DHC) and often low serum total cholesterol. There is a broad range of cholesterol seen in SLOS (less than 10 mg/dL to greater than 200 mg/dL). It remains uncertain whether morphologic and behavioral manifestations of SLOS are caused by decreased cholesterol levels, increased 7-DHC, or both. SLOS is associated with ASD in 50–75% of cases [6, 18, 19]. To date, the neurobiologic relationship between SLOS and ASD has not been explained. 

**Figure 2:**
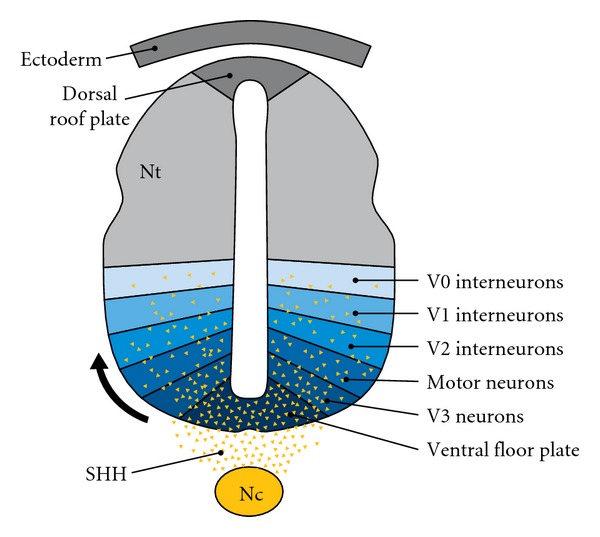
The sonic hedgehog gradient in embryonic neural patterning. SHH-regulated gradient defines neuronal subtypes during embryonic patterning. Sonic hedgehog (SHH) (yellow) is secreted from cells of notochord (Nc) and ventral floor plate to create a ventral-dorsal concentration gradient along the neural tube (Nt). Spatial organization of six progenitor-cell domains is established by the SHH gradient restricting the expression of various protein-marker profiles. The initiation of these markers at successive developmental time periods results in V0–V3 and motor neuron (MN) subtype patterning along the ventral midline in the neural tube.

Sonic Hedgehog (*SHH*) is a morphogen involved in the patterning of the nervous system and limbs, along with other transcription factors and secreted proteins [20–25]. During embryonic development, *SHH* is covalently modified with both palmitate and cholesterol and secreted as part of a lipoprotein complex that regulates brain morphogenesis through the patched/smoothened signaling system [26–29]. *SHH* is secreted from the notochord and ventral floor plate cells and forms a concentration gradient along the entire dorsal-ventral axis [29]. The posttranslational effect of *SHH* after covalent modification by cholesterol is the establishment of a morphogenic *SHH *concentration gradient that moves from the ventral (high concentration) to dorsal regions (lower concentration). Variations in the *SHH* gradient affect intracellular cell signaling systems and ultimately determine the expression of future cell types by sequential induction of transcription factors in ventral progenitor cells [29]. The formation of discrete cell precursor domains in the neural tube as a result of the *SHH* morphogenic front is one determinant of the structural fate of the maturing brain [30–32] (Figure 2). In animal studies, during late embryonic and postnatal brain development, neural precursor and stem cell proliferation in dorsal neocortical, hippocampal, tectal, and cerebellar regions is regulated by *SHH *signaling [33, 34]. In humans, failure of midline brain structures to form appropriately can result from a loss of *SHH* processing, as evidenced in holoprosencephaly [35]. Incomplete formation of midline structures including the corpus callosum and cerebellum is the most common neuroimaging abnormality found in individuals with SLOS [36]. Interestingly, reduction in corpus callosum size is among the most common neuroimaging abnormality in autism and supports the aberrant connectivity hypothesis that autism is a disorder of connectivity, involving inter- and intrahemispheric communications with possible alterations of intracortical connections [37–39]. In both autism and SLOS, it is uncertain whether callosal hypoplasia is due to a primary patterning defect or later dysfunction of neuronal cortical connectivity and axonal migration or both. 

We hypothesize that in SLOS, low cholesterol or elevated sterol precursors result in establishment of an abnormal *SHH* gradient, which may alter the fate of cells in the developing brain. Further studies are required to support this hypothesis. While the hypothesis may be plausible for SLOS and certain cholesterol-dependent ASD, incomplete formation of midline structures is present in numerous disorders of cognition and behavior without abnormal sterol biosynthesis. In addition, there are many individuals with ASD that do not have midline structural brain abnormalities. For these reasons, multiple mechanisms are likely to arise as etiologies of the ASD phenotype. In sum, regional differences in the establishment and advancement of the *SHH* gradient and its effects on transcription factors, may provide an explanation for the development of cognitive and behavioral impairment in disorders with diffuse neural abnormalities, such as autism and SLOS.

## 3. Membrane Lipid Rafts and ASD

Studies on cholesterol and lipid organization in disease have led to progress in understanding the molecular basis of neurologic disorders [40]. As a result, autism research involving sterols and other metabolites continues to gain popularity. For over a decade, lipid rafts or specialized membrane microdomains have been investigated for their key role in cellular communication [41, 42]. Rafts are dynamic structures enriched with cholesterol, sphingomyelin, and phosphatidylcholine [43]. The primary raft subtype called caveolae comprised of scaffolding proteins (caveolin), is distinguished by flask-shaped invaginations of the plasma membrane [44]. These platforms serve as signaling regions in clatharin-independent endocytosis, lipid homeostasis, signal transduction, and tumorigenesis [45]. Caveolae are widely expressed in brain endothelial cells, astrocytes, oligodendrocytes, Schwann cells, dorsal root ganglia, and hippocampal neurons [46]. Lipid rafts play a critical role in many neurologic disorders including SLOS, Huntington disease, Alzheimer's disease, Tangier disease, and Niemann-Pick disease type C [40, 47, 48]. The essential role of cholesterol in formation of lipid rafts and membrane organization is highlighted in studies of membrane physiology. Cholesterol content is extremely important for cell membrane lateral organization and protein function [49–51]. Samuli Ollila et al. [49] report that lipid membrane lateral pressure profiles were significantly altered when cholesterol was replaced with sterol precursors, desmosterol, 7-DHC, or ketosterol. Furthermore, 7-DCH and 8-DHC have been shown to accumulate in membrane lipid rafts of liver tissue in individuals with SLOS [52]. The accumulation of sterol precursors in rafts depletes cholesterol from structures such as hippocampal membranes and limits ligand-binding activity of the serotonin 1A receptor [53]. Functional changes at the cellular level may be explained by studies showing that *DHCR7*-deficient neuronal cell lines downregulate genes critical to lipid synthesis such as sterol-regulatory element binding protein 2 (SREB-2), SREBF chaperone, site-1 protease, fatty acid synthase, and squalene synthase [47]. Decreased *DHCR7* has also been shown to alter expression of key molecules for intracellular signaling and vesicular transport such as Egr1, Snx, and Adam19 [47]. These studies support a possible role for abnormal neuronal cell membrane protein signaling in *DHCR7* mutations that lead to behavioral manifestations in SLOS. More studies are needed to determine if these mechanisms are involved in the human pathophysiology of SLOS and other neurodevelopmental disorders. Rafts may represent one of the many biologic substrates that shape neuronal networks in the brain. Recent data has shown that reduction in cholesterol levels impair exocytosis of synaptic vesicles [54]. Numerous questions are surfacing about the clinical manifestations of neuronal and glial membrane alterations caused by altered lipid raft composition in humans. For example, it remains unknown whether membrane proteins important for synaptic plasticity such as AMPA kainate, GABA_A_, and NMDA receptors are affected by abnormal sterol levels or whether these abnormalities are present either transiently or for longer periods in regions of the developing brain for individuals with autism. Therefore, we hypothesize that neuronal or glial expression of autism candidate genes and their resulting membrane proteins may be altered in disorders of abnormal cholesterol homeostasis.

## 4. Neurosteroids and ASD

Neurosteroids are steroid molecules produced by the central nervous system to rapidly augment neuronal excitability through membrane-bound, ion-gated neurotransmitter receptors [55, 56]. While classic steroid hormones typically exert endocrine function on the order of hours to days, neuroactive steroids can act rapidly in a nontranscriptional mechanism to produce behavioral effects in seconds to minutes [56–59]. Neuroactive steroids are synthesized from cholesterol in neurons and glia or steroid precursors from peripheral tissues [60, 61]. Expression of steroidogenic enzymes is developmentally regulated [62]. There are many different types of neurosteroids resulting in an array of functional diversity including positive allosteric modulation of GABA_A_ and NMDA receptors, myelin formation, axonal guidance, and dendrite growth [55, 62, 63]. These molecular activities enable moment-to-moment modulation of neuroendocrine functions and behavior.

Because of their broad psychiatric characteristics, neurosteroids have been implicated in the behavioral profile of SLOS [64]. Biochemical studies have demonstrated that neurosteroids possess pharmacologic properties applicable to anesthesia and epilepsy [57, 65]. Benzodiazepines inhibit the enzymes responsible for neurosteroid metabolism, perhaps due to shared pharmacologic action at the GABA_A_ receptor [66]. Interestingly, some antidepressant agents such as fluoxetine have been found to increase circulating neurosteroid levels [67, 68]. The molecular effects of these medications on the nervous system in SLOS have not been investigated.

Since cholesterol does not cross the blood-brain barrier, neurosteroids are synthesized with cholesterol de novo [69]. For nearly a decade, it has been proposed that increased 7-DHC levels might inhibit neurosteroid formation or lead to synthesis of an inhibitory analog in the brain [70]. Marcos et al. [64] studied urinary steroids and found that dehydrocholesterols provided the substrate for formation of allopregnanolone and dehydroallopregnanolone in patients with SLOS. While only providing evidence for extraneural synthesis of 7- and 8-dehydroallopragnanolones, there is a high likelihood that abnormal synthesis occurs in the brain given the low tissue specificity of 5*α*-reductase and 3*α*-hydroxysteroid dehydrogenase [64]. Currently, mouse model studies are investigating the prospect that reduced levels of neurosteroids possessing anxiolytic properties, such as allopregnanolone, impact behavior in SLOS.

## 5. Targeted Therapeutics and Conclusions

Current treatment of SLOS involves endogenous cholesterol supplementation in the form of crystallized purified cholesterol suspended in Ora-Plus, microencapsulated powdered purified cholesterol (brandname SLOesterol), or egg yolks. Several publications discuss the role of simvastatin therapy [71–73]. Efficacy for either of these therapies remains unclear. Endogenous cholesterol biosynthesis is the primary mechanism for nervous system cholesterol homeostasis, making a role for extrinsic cholesterol in altering nervous system function questionable [47]. As we look ahead, pharmacologic agents derived from neuroactive steroids or steroid analogues may provide targeted therapy for behavioral symptoms in SLOS and ASD. Currently, clinical trials are examining the therapeutic effects of neurosteroids on mood disorders, schizophrenia, substance abuse, traumatic brain injury, and cognitive disorders. Lipids such as 7-DHC may undergo perioxidation to form bioactive products called oxysterols that have been shown to reduce proliferation of Neuro2a cells and induce cell differentiation [74]. Oxysterols have long been hypothesized in the pathology of SLOS and remain a promising area for interventional trials to reduce oxygen free radicals [75–78]. Enzyme-specific candidate drugs are being investigated in SLOS. Appropriate modulation of embryonic *SHH* patterning and lipid rafts are not likely to be achieved until future studies elucidate the specific mechanisms and biologic substrates underlying brain development. These studies may be aided by advances in functional neuroimaging and molecular imaging techniques. Furthermore, discussion on the ethics involving embryologic or childhood neuromodulatory therapy in patients with abnormal neural patterning should be considered if technology advances toward such a therapeutic option. In conclusion, we propose that ASD in SLOS, and perhaps other disorders of cholesterol homeostasis, occurs because of impairments in sonic hedgehog patterning, altered lipid raft structure resulting in aberrant synaptic plasticity, and impaired neuroactive steroid synthesis. Future investigations to explore these hypotheses are encouraged and may enhance our understanding of sterols in autism and other neurodevelopmental disorders.
